# Microfluidic-based fabrication and characterization of drug-loaded PLGA magnetic microspheres with tunable shell thickness

**DOI:** 10.1080/10717544.2021.1905739

**Published:** 2021-04-05

**Authors:** Chunpeng He, Wenxin Zeng, Yue Su, Ruowei Sun, Yin Xiao, Bolun Zhang, Wenfang Liu, Rongrong Wang, Xun Zhang, Chuanpin Chen

**Affiliations:** a Xiangya School of Pharmaceutical Sciences, Central South University, Changsha, China; b Hunan Zaochen Nanorobot Co., Ltd, Liuyang, China; c Affiliated Haikou Hospital of Xiangya Medical College, Central South University, Haikou, China; d Hunan Institute for Drug Control, Changsha, China

**Keywords:** TACE, microfluidic, microsphere, paclitaxel

## Abstract

To overcome the shortcoming of conventional transarterial chemoembolization (cTACE) like high systemic release, a novel droplet-based flow-focusing microfluidic device was fabricated and the biocompatible poly(lactic-co-glycolic acid) (PLGA) magnetic drug-eluting beads transarterial chemoembolization (TACE) microspheres with tunable size and shell thickness were prepared via this device. Paclitaxel, as a model active, was loaded through O/O/W emulsion method with high efficiency. The size and the shell thickness vary when adjusting the flow velocity and/or solution concentration, which caters for different clinical requirements to have different drug loading and release behavior. Under the designed experimental conditions, the average diameter of the microspheres is 60 ± 2 μm and the drug loading efficiency has reached 6%. The drug release behavior of the microspheres shows the combination of delayed release and smoothly sustained release profiles and the release kinetics differ within different shell thickness. The microspheres also own the potential of magnetic resonance imaging (MRI) visuality because of the loaded magnetic nanoparticles. The microsphere preparation method and device we proposed are simple, feasible, and effective, which have a good application prospect.

## 1. Introduction

Hepatocellular carcinoma (HCC), the sixth most common cancer incident cases, is the most frequent primary liver cancer and ranks as the fourth leading cause of cancer-related death around the world (Parkin et al., 2005; Torre et al., 2015; Bray et al., 2018). The World Health Organization once estimated that over 1 million patients would die from liver cancer in 2030 (Villanueva, 2019). Heavily favored staging strategy for HCC currently is Barcelona Clinic Liver Cancer (BCLC) staging system, which includes four disease stages (early, intermediate, advanced, and terminal) (Piscaglia & Bolondi, 2010). Resection, liver transplantation, and percutaneous ablation are three available options for the treatment of early-stage HCC; however, only a minority of HCC patients (less than 20%) are included (Zhao & Ma, 2019) because exceeding 80% of patients are diagnosed as the intermediate and terminal cancer (Liang et al., 2020). For these patients, transarterial chemoembolization (TACE) is the treatment of choice. TACE is a minimally invasive procedure widely performed globally. Some small embolic materials are injected into the artery directly supplying the tumor. The embolic materials would block the blood and nutrition supply, which will cause tumor necrosis (Nakamura et al., 1989). TACE has become the gold treatment standard for HCC patients in intermediate stage and it has been chosen as the bridge therapy to liver resection or transplantation, which shows its broad prospects (Coletta et al., 2017).

Conventional TACE (cTACE) includes the utilization of agents like lipiodol, chemotherapeutics, and embolic materials. Lipiodol acts as the carrier of chemotherapeutics drugs deposited in the tumor and other embolic materials will prevent the rapid washout of chemotherapeutic agent. However, the motility of lipiodol will reduce the concentration of chemotherapeutics agents, and drug release behavior cannot be controlled, which will affect the treatment result (Song & Kim, 2017). Drug-eluting beads (DEBs) TACE was introduced in 2006 aiming at overcoming the drawbacks of cTACE and it has become an effective alternative (Lewis et al., 2006). It uses the polymer microspheres as drug-carrier and embolic materials. After the delivery of drug-loaded polymer microspheres to certain place, chemicals like doxorubicin or epirubicin encapsulated would release as sustained characters. Compared with cTACE, DEB-TACE can reduce the systemic exposure of chemotherapeutic agent and enlarge the concentration of agents of the tumor region (Melchiorre et al., 2018). Due to the advantages above, several commercially available DEB-TACE microspheres such as CalliSpheres^®^ Beads, DC Beads^®^, Contour SE^®^ microspheres, and HepaSphere Microspheres^®^ are approved for clinical use (Patel et al., 2005; Song et al., 2013; Malagari et al., 2014; Wu et al., 2018).

DEB-TACE has strict requirements on the size and size distribution of the microspheres because only using the homogeneous size particles can ensure the particles localize and embolize the arterial as the predictable way and the drugs encapsulated release in a predictable process. What is more, in order to enhance the embolization effect, it is of great necessary to prepare microspheres with tunable size to fit different requirements of treatments on different animals or organs. Traditional microspheres fabrication approaches like phase separation and emulsifying solvent evaporation utilize inhomogeneous forces in the synthesis procedures. Hence, these methods tend to result in the production of microspheres with broad size distribution and high initial burst release, leading to the unreliable and unpredictable drug release kinetics and drug delivery efficiency, which cannot meet the complicated requirements of DEB-TACE. Besides, adjusting the experiment parameters to change the size and size distribution is time-consuming and resource intensive. Droplet-based microfluidic technology, an alternatively promising approach, has been gradually used to produce polymeric nanoparticles (NPs), microspheres, and microbubbles because of the capacity of generating highly uniform and monodisperse droplets (Prasad et al., 2009; Breslauer et al., 2010; Gong et al., 2019). Based on the traditional microfluidic system, the droplet-based microfluidic technology utilizes two fluids or more, which cannot dissolve each other, to produce the micro-scale droplet by the synergy of surface tension and shear force. By adjusting the flow velocity or velocity ratio of these fluids, we can prepare the microspheres with different sizes or different structures, which lead to different drug release kinetics.

Lipiodol is currently used as the contrast agent in TACE treatment because of its excellent X-ray imaging capacity (Cao et al., 2019). However, the radiation of X-ray poses a potential hazard to human health, and the lipiodol embolized into the tumor tends to diffuse among the body because of the liquid motility, which will cause severe lipiodol embolism (Xu et al., 2014; Tan et al., 2019). Magnetic resonance imaging (MRI) technology has become an alternative of X-ray imaging in recent years. MRI utilizes the MR signals created during the relaxation of hydrogen proton which resonated with the radio waves under strong magnetic fields. Compared with X-ray imaging, MRI can track the location of embolic chemicals real time without the radiation hazards (Cilliers et al., 2008; Yu et al., 2009; Choi et al., 2015). Hence, MRI can be seen as a better choice than X-ray imaging.

Here, we presented a novel microspheres preparation platform with the flow-focusing microfluidic chips derived from droplet-based microfluidic technology. Magnetic nanoparticles (MNPs) and chemotherapeutic drug co-encapsulated microspheres were prepared via this device. Poly(lactic-co-glycolic acid) (PLGA) is a sort of biodegradable polymer approved by the United States Food and Drug Administration and the European Medicines Agency as reliable excipients (Jain, 2000) and a majority of formulations have been focused on the PLGA as its excellent drug-load and sustained release capacity (Hung et al., 2010; Obayemi et al., 2016; Bokharaei et al., 2017; Li et al., 2017; Nanaki et al., 2017). MNPs were introduced to enhance the MRI visuality. Paclitaxel (PTX), as a model active, was loaded through O/O/W emulsion method with high efficiency. We also systematically demonstrated the influence of flow rate on the shell thickness of PLGA microspheres, which affects the rate of drug release. The multi-functional microspheres prepared can realize the joint function of embolism therapy and chemotherapy in addition to the potential of MRI imaging, thus improve the efficacy and the prognosis effect of TACE.

## 2. Experimental

### 2.1. Materials

PLGA (copolymer 50:50, Resomer RG503, MW = 43,000 Da) was supplied by Shandong Institute of Medical Instrument (Zibo, China). Sylgard 184 silicone elastomer kits including polydimethylsiloxane (PDMS) and the curing agent were purchased from Dow Corning Co., Ltd. (Midland, MI). Poly(vinyl alcohol) (PVA) was purchased from Tianjin Kemel Chemical Reagent Co., Ltd. (Tianjin, China). Paclitaxel was purchased from Shanghai Yuanye Bio-Technology Co., Ltd. (Shanghai, China). Other chemicals were of analytic grade and purchased from Sinopharm Chemical Reagent Co., Ltd. (Shanghai, China).

### 2.2. Synthesis of Fe_3_O_4_ nanoparticles

The Fe_3_O_4_ NPs were synthesized with the chemical coprecipitation method of Fe^2+^ and Fe^3+^ salts (Arruebo et al., 2007). Briefly, FeCl_2_·4H_2_O and FeCl_3_·6H_2_O were separately dissolved in deoxidized deionized water and mixed in a ferric ion ratio of 1:2 (Fe^2+^/Fe^3+^, *C*_Fe2+_=0.01 M) under vigorous stirring. The solution turned into black gradually by adding NH_4_OH dropwise to pH 11.0 under nitrogen gas protection, followed by 1.0 mL oleic acid (OL) tricked to the mixture solution. When the solution was heated to 75 °C, vigorous stirring was continued for another 60 minutes under a stream of nitrogen. The Fe_3_O_4_ NPs prepared were washed for five times with deionized water. The NPs were dried into powders by oven before usage.

### 2.3. Fabrication of PDMS microfluidic devices

**Figure 1.**
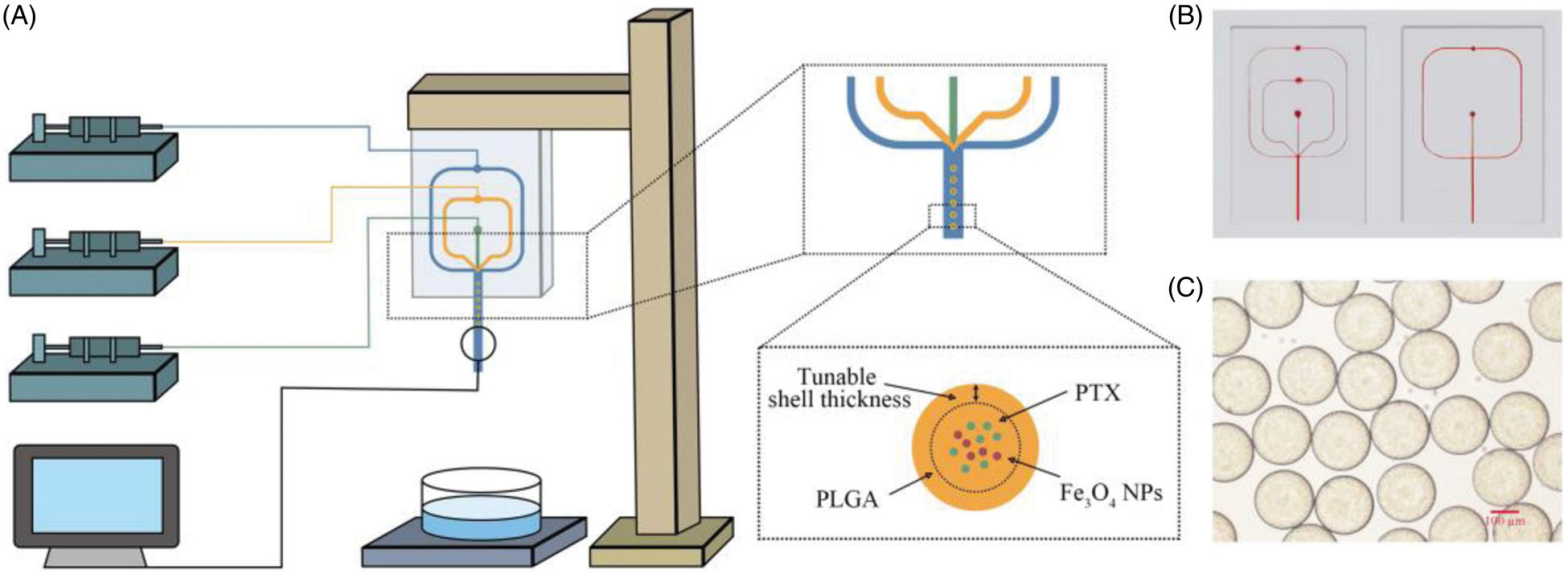
The emulsion droplet generation microfluidic device for the preparation of PLGA microspheres. (A) Schematic illustration of the three-phase flow-focusing microfluidic set up. Three syringe pumps control the fluid injected through the chip. The droplets were collected into a glass dish. (B) Photographs of three-phase (left) and two-phase (right) microfluidic chips. (C) Optical photograph of PLGA microdroplets prepared via this microfluidic device.

As shown in Figure 1(B), two types of microfluidic devices with different channel geometries were used to produce PLGA microspheres. One of them was a three-phase flow-focusing microfluidic device and the other was a two-phase one. The widths of the inlet channel and the cross-sectional dimensions channel are 100 μm and 350 μm, respectively. The height of the channel is 100 μm.

The flow-focusing PDMS based microfluidic chip was fabricated by using the standard photolithographic and soft lithography processes (Peng et al., 2019). The transparency mask was designed with Adobe Illustrator (CS 6, Adobe System Co., Ltd., San Jose, CA) and printed by high-resolution printer. Four layers of dry film (25 μm, Riston FX900, DuPont Co., Ltd., Wilmington, DE) was stuck on the polyethylene terephthalate (PET) film (0.175 mm, Zhuhai Kaivo Optoelectronic Technology Co., Ltd., Zhuhai, China) and exposed to ultraviolet (UV) (HT-3040, Shijiazhuang Ourpcb Co., Ltd., Shijiazhuang, China) for 120 s with the transparent photomask. After being exposed, the dry film was developed in 1 wt.% CaCl_2_ solution for 10 min. The mold was fabricated after washing and drying the dry film stuck PET film. Sylgard 184 curing agent and pre-polymer were mixed with 1:10 ratio and poured on the microfluidic chip mold. After degassing, the mixture was cured at 60 °C for 2 h (Wang et al., 2016). The surface of the PDMS was OH-terminated by a plasma treatment (TW-2K, Ruian Zhilin Technology Co., Ltd., Ruian, China) and bound with a plasma treated PDMS cover slide. The PDMS device was then baked at 60 °C (101-0AB, Tianjin Taiste Instrument Co., Ltd., Tianjin, China) for 2 h to get closer integration. Glass capillaries (250 μm i.d. 350 μm o.d., TSP250350, Polymicro Technologies Co., Ltd., Phoenix, AZ) were sealed to the outlet of the microchannels. The inlet of the chip was connected to the syringe pumps through the polytetrafluoroethylene (PTFE) tube (0.5 mm i.d. 1.0 mm o.d., Ruixia Co., Ltd., Shanghai, China).

### 2.4. Preparation of magnetic PLGA microspheres using the flow-focusing microfluidic device

We used the two-phase microfluidic flow-focusing device to investigate the effects of experimental parameters on the size and size distribution. The outer phase was composed of 1 wt.% PVA aqueous solution and the dichloromethane (DCM) solution of PLGA containing varied amount of Fe_3_O_4_ NPs was chosen as inner phase. All these streams were driven by syringe pumps (LSP01-1A, Longer Precision Pump Co., Ltd., Baoding, China) independently. The inner phase was sheared by the outer phase at the flow-focusing intersection and the O/W emulsion droplets formed. Subsequently, the droplets were collected with aqueous solution of 1 wt.% PVA. The flow and the droplet formation process were monitored by a stereomicroscope (GP-660V, Gaopin Co., Ltd., Beijing, China). The suspension was stirred by the mechanical stirrer for 6 h to evaporate DCM. The residual was centrifuged at 5000 rpm for 10 min (TGL-16, Yingtai Instrument Co., Ltd., Changsha, China) and then washed for four times with deionized water to remove the redundant PVA. The morphology and size were captured by inverted fluorescence microscope (ECLIPSE Ti, Nikon Co., Ltd., Tokyo, Japan) with bright field and calculated by Image J (2.0.0, National Institutes of Health, Bethesda, MD).

### 2.5. Drug loading

As anticancer drugs, PTX is commonly used clinically and the PTX-loaded magnetic PLGA microspheres were prepared in O/O/W emulsion method with the three-phase microfluidic flow-focusing device. The schematic illustration of experiment set up and droplet formation is shown in Figure 1. Briefly, 1 wt.% PVA aqueous solution and 1 wt.% PLGA DCM solution containing varying amount of PTX and Fe_3_O_4_ NPs were chosen as outer and inner phase, respectively. The intermediate phase consisting of 1 wt.% PLGA was used as an obstacle which hindered the leakage of PTX. In order to evaluate the block effect of intermediate phase, four sets of parameters were chosen to evaluate the drug loading capacity and release kinetics profiles (Table 1). They shared the same total flow velocity but different flow rate of intermediate phase and inner phase. When the flow velocity of intermediate phase is 0 μL/min, we replaced the three-phase microfluidic flow-focusing device with a two-phase one. To ensure particles of each group had the same effects, we adjusted the concentration of Fe_3_O_4_ NPs and PTX to make these particles own the same number of contents. After solvent evaporation and solidification, the microspheres were washed for four times by deionized water to remove the redundant PVA. All these microspheres were lyophilized (LC-10N-80A, Lichen Co., Ltd., Shanghai, China) for 24 h and stored at 4 °C for further studies.

**Table 1.** Drug loading capacity of PTX-loaded magnetic microspheres formulations with different velocity ratio of outer, intermediate, and inner phase.

Group	Flow rate (μL/min)	Concentration of Fe_3_O_4_ (mg/mL)	Concentration of PTX (mg/mL)	Drug loading rate (%)	Encapsulation efficiency (%)
Group 1	500/0/60	0.33	1	5.41 ± 0.41	59.71 ± 4.40
Group 2	500/10/50	0.40	1.2	6.52 ± 0.43	67.35 ± 4.41
Group 3	500/30/30	0.67	2	6.98 ± 0.33	72.11 ± 3.40
Group 4	500/50/10	2.00	6	6.82 ± 0.11	70.45 ± 1.13

### 2.6. Characterization

The mean diameter and polydispersity index (PDI) of Fe_3_O_4_ NPs were measured by particle size analyzer (Zetasizer Nano ZS 90, Malvern Co., Ltd., Malvern, UK). An inverted fluorescence microscope with bright field was used to capture the morphology of the wet PLGA microspheres. The average size and size distribution were evaluated by Image J (National Institutes of Health, Bethesda, MD). Fourier transform infrared spectra (FTIR) of Fe_3_O_4_ NPs, PLGA polymer, PTX, and the microspheres were recorded by a FTIR spectrometer (8300, Shimadzu Co., Ltd., Kyoto, Japan). The pellet was prepared using KBr method.

### 2.7. Drug loading capacity and drug release *in vitro*

Ten milligrams freeze-dried PTX-loaded microspheres were solubilized in methanol with high intensity sonication. The solution was filtered through a 0.22 μm filter (Jinteng Laboratory Equipment Co., Ltd., Tianjin, China) and the filtrate was analyzed by high-performance liquid chromatography (HPLC, Acquity Arc, Waters Co., Ltd., Milford, MA) with the UV detector at the wavelength of 229 nm. The drug loading rate (LC) was calculated by the following equation:

(1) $$\mathrm{LC}\;(\%)=\frac{W_{1}}{W_{2}}\times 100$$

where $W_{1}$ and $W_{2}$ represent the weight of drug in microspheres and feeding drug, respectively.

The encapsulation efficiency (EE) was calculated by the following equation:

(2) $$\mathrm{EE}\;(\%)=\frac{a\text{ctual}\text{ LC }(\%)}{t\text{heoretical}\text{ LC }(\%)}\times 100$$

The release kinetics profiles were quantified by HPLC-UV. Ten milligrams PTX-loaded microspheres were placed in pretreated dialysis membrane (MW = 3500 Da), which was immersed in 20 mL phosphate buffer solution (PBS, 0.01 M, pH 7.2). The temperature and the rotation rate were maintained by incubator (37 °C, 100 rpm, SPX-100B-D, Boxun Industry & Commerce Co., Ltd., Shanghai, China). At certain time intervals, 1 mL PBS medium was extracted, and isometric fresh medium was added. PBS extracted was filtered through a 0.22 μm filter. The samples were injected to HPLC.

## 3. Results and discussion

### 3.1. Characterizations of Fe_3_O_4_ nanoparticles

**Figure 2.**
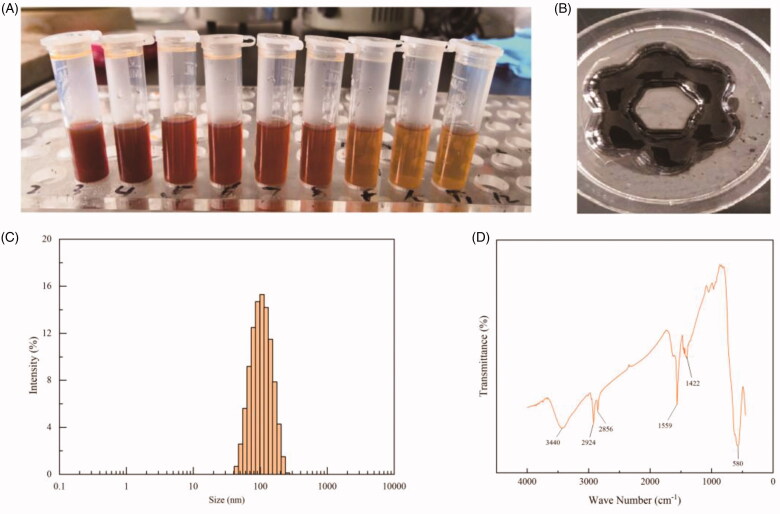
Characterizations of Fe_3_O_4_ nanoparticles prepared with chemical coprecipitation method. (A) Photographs of Fe_3_O_4_ nanoparticles suspensions with different concentrations. The concentrations are 2, 1.33, 1, 0.8, 0.67, 0.55, 0.44, and 0.4 mg/mL respectively from left to right. (B) Photographs of Fe_3_O_4_ nanoparticles in the presence of a flower petal shaped magnet. (C) Size distribution and (D) FTIR spectrum of oleic acid bonded Fe_3_O_4_ nanoparticles.

**Figure 3.**
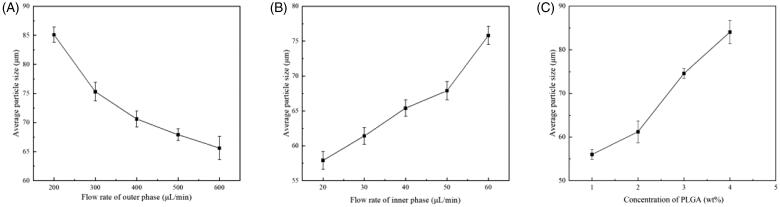
The influence of applied parameters on the size of microspheres. (A) The effect of flow rate of outer phase. The flow rate of inner phase is 50 μL/min constant. The concentrations of Fe_3_O_4_ NPs and PLGA are 1 mg/mL and 1 wt.%, respectively. (B) The effect of flow rate of inner phase. The flow rate of outer phase is 500 μL/min constant. The concentrations of Fe_3_O_4_ NPs and PLGA are 1 mg/mL and 1 wt.%, respectively. (C) The effect of concentration of PLGA. The concentration of Fe_3_O_4_ NPs is 1 mg/mL and the velocity ratio of inner and outer phase is 20/300.

The Fe_3_O_4_ NPs with different concentrations had good stability and exhibited excellent magnetic properties when approaching to a magnet (Figure 2(A,B)). The NPs possessed monodispersed spherical shapes with a mean diameter of 94.49 nm (PDI = 0.119, Figure 3(C)). The FTIR spectrum of Fe_3_O_4_ NPs showed that the peaks at 1559 cm^−1^ and 1422 cm^−1^ correspond to the symmetric and antisymmetric stretching vibration of the oleate, and the peak at 580 cm^−1^ to the Fe–O bond (Figure 2(D)). These results confirmed that the OL has been bonded to the surface of Fe_3_O_4_ NPs.

### 3.2. Preparation of magnetic PLGA microspheres using the flow-focusing microfluidic device

DEB-TACE microspheres should have homogeneous size to make sure the particles would localize and embolize the arterial as the predictable way. Hence, we investigated the size and morphology of the microspheres. A two-phase microfluidics flow-focusing device was designed to generate the microspheres. The microfluidic device designed consists of a flow focusing channel, an outlet and two inlets, in which the flow streams of inner phase were sheared into small droplets by outer phase. The flow rates of outer phase and inner phase, named *Q*_o_ and *Q*_i_, respectively, were investigated to form droplets of different sizes. Inverted fluorescence microscope with bright field was chosen to capture the morphology of the wet microspheres. The result indicated that the particle size decrease with the increase of *Q*_o_ and the decrease of *Q*_i_ (Figure 3(A,B)). What is more, the concentration of Fe_3_O_4_ NPs and PLGA would make a tremendous difference to the size and size distribution. Figure 3(C) shows that the size increased when increasing the concentration of PLGA, which may because a higher concentration leaded to a higher viscosity, and the latter is an important factor for microsphere preparation. However, the high-viscosity fluid would increase the risk of micro-channel blocking.

### 3.3. Preparation of PTX-loaded magnetic PLGA microspheres

**Figure 4.**
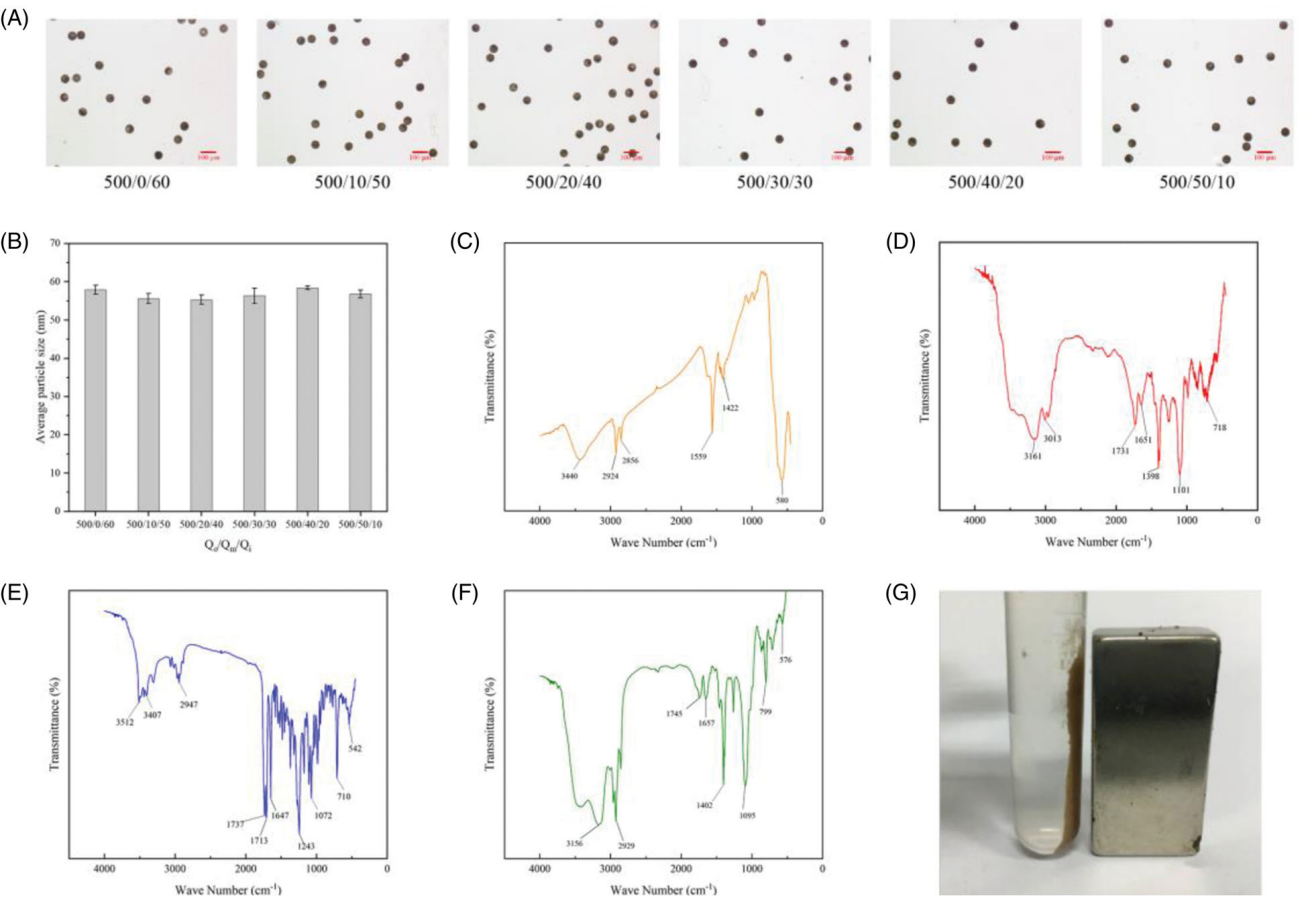
Characterization of PTX-loaded magnetic PLGA microspheres. (A) Optical photographs of PTX-loaded magnetic PLGA microspheres prepared with different flow velocities (*Q*_o_/*Q*_m_/*Q*_i_). (B) The size analysis results of the optical photograph. (C–F) The FTIR spectra of oleic acid bonded Fe_3_O_4_ nanoparticles (C, orange), PLGA (D, red), PTX (E, blue), and PTX-loaded magnetic PLGA microspheres (F, green). (G) Photograph of PTX-loaded magnetic PLGA microspheres in the presence of a magnet.

In this study, we also precisely controlled the size of microspheres by employing different flow rate of outer phase (*Q*_o_), intermediate phase (*Q*_m_), and inner phase (*Q*_i_). We found that when holding the overall flow velocity and *Q*_o_ but adjusting the *Q*_i_ and *Q*_m_, the size showed no significant difference (Figure 4(A,B)). Basing on these, we designed four groups of parameters to prepare microspheres. The overall flow velocities and *Q*_o_ are the same but the *Q*_i_ and *Q*_m_ are different, which means the *Q*_i_ and *Q*_m_ were adjusted on the condition that the sum of *Q*_i_ and *Q*_m_ is constant. After solidification, the intermediate phase became the shell to prevent the leakage of PTX. Different Q_m_s signify different thicknesses of the PLGA shell, which will result in different hinder effects. The microscopy images indicated that microspheres in these four groups were entirely spherical and uniform in size. Besides, they shared similar particle size of 60 ± 2 µm, which is because the flow velocity altogether is constant (Figure 4(A,B)).

To investigate the composition of the PLGA microspheres, FTIR was conducted and the spectrum was compared with Fe_3_O_4_ NPs, PLGA, and PTX. The spectra for microspheres indicated the presence of Fe_3_O_4_ NPs (576 cm^−1^). The appearance of prominent peak at 1745 cm^−1^ confirmed the COO bond of PLGA and the wide peak at 3500–3450 cm^−1^ was corresponded to carboxylic acid of PLGA. The FTIR spectrum of microspheres showed all the characteristic peaks of PLGA and Fe_3_O_4_ NPs but no PTX signals owing to low concentration of PTX in microspheres (Figure 4(C–F)). Besides, the PTX loaded magnetic PLGA microspheres also presented mobility under the external magnetic field applied (Figure 4(G)).

### 3.4. Drug loading capacity and drug release *in vitro*

When the weight ratio of PTX and PLGA was 1:10, the drug loading capacity differed within the four groups (Table 1). The microspheres with a thicker shell (group 3 and group 4) showed a larger loading capacity than a thinner one (group 1 and group 2), which because the shell could prevent the diffusion of PTX.

**Figure 5.**
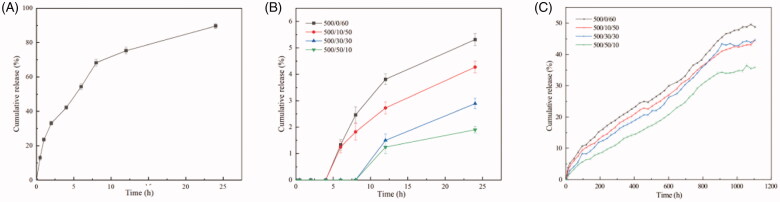
Release profiles of free PTX (A) and PTX-loaded magnetic microspheres (B, C) with different preparation parameters. The flow rate of outer phase was 500 μL/min constantly and the velocity ratio of intermediate and inner phase is 0/60 (black line), 10/50 (red line), 30/30 (blue line), 50/10 (green line), respectively.

Figure 5 shows the cumulative release curves of PTX from these four-group microspheres, respectively. The microspheres prepared are applied in TACE treatment. In TACE procedure, the microspheres are finally located into artery and block the blood vessels. Hence, the microspheres are immersed in blood. In order to ensure the consistency of drug release *in vitro* and *in vivo*, 0.01 M PBS was chosen as the release medium because its pH value and osmotic pressure are similar to blood. *In vitro* drug release profiles showed that the primarily release occurred within in the first 2 h and all these microspheres exhibits sustained release profiles. About 20–30% PTX was released after 25 days of incubation. The cumulative release percentage of microspheres with thicker shell were slightly lower than that with a thinner one. This phenomenon can be attributed to the inhibition of PLGA shell. Besides, all groups showed no burst release but different levels of smoothly sustained release and delayed release characteristics. By adjusting the time window of delayed release and release rate, we can achieve different drug release kinetics, which applying to complex clinical applications.

To expound the mechanism of these drug release profiles, zero-order equation, first-order equation, the Korsmeyer–Peppas equation, and Higuchi’s equation were used to fit the release data. As listed in Table 2, the Korsmeyer–Peppas equation fits best among all the models used and the correlation coefficient is 0.9918 in the Korsmeyer–Peppas equation, release coefficient *n* is an important factor, showing the mechanism of drug release. The release coefficients in these four groups are 0.8764, which means the drug was released in diffusion and erosion mechanism.

**Table 2.** Fitting result of PTX-loaded magnetic microspheres with different shell thickness on the zero-order equation, first-order equation, Higuchi’s equation, and the Korsmeyer–Peppas equation.

Group	Zero-order equation		First-order equation		Higuchi’s equation		The Korsmeyer–Peppas equation		
	$\frac{Q_{t}}{Q_{\infty }}=k_{o}$		$\frac{Q_{t}}{Q_{\infty }}=1-e^{-k_{1}t}$		$\frac{Q_{t}}{Q_{\infty }}=k_{H}t^{1/2}$		$\frac{Q_{t}}{Q_{\infty }}=k_{t}t^{n}$		
	*k*_0_	*R* ^2^	*k*_1_	*R* ^2^	*k_H_*	*R* ^2^	*k_t_*	*n*	*R* ^2^
Group 1	0.0009	0.9898	0.0044	0.5075	0.0338	0.9630	0.1017	0.8764	0.9918
Group 2	0.0009	0.9899	0.0029	0.8860	0.0327	0.9709	0.1017	0.8764	0.9918
Group 3	0.0009	0.9890	0.0036	0.7931	0.0347	0.9527	0.1017	0.8764	0.9918
Group 4	0.0009	0.9881	0.0032	0.8522	0.0373	0.9481	0.1017	0.8764	0.9918

## 4. Conclusions

In this study, a simple flow-focusing microfluidic device was fabricated and the uniform and monodisperse magnetic PLGA microspheres were prepared. Anticancer drugs PTX, as a model chemical, was loaded into the microspheres using the O/O/W emulsion method. With this device, the size and shell thickness can be preciously controlled, which caters for the complex requirements of clinical applications. The PTX-loaded magnetic PLGA microspheres exhibited delayed release and smoothly sustained release kinetics without burst release phenomenon. The magnetic microspheres presented mobility under the external magnetic field applied, which showed its excellent magnetic response capacity. Our study has presented the microsphere preparation method and demonstrated its advantages in preparing highly uniform microspheres and the ability to control release kinetics. Therefore, the application potential of such a method and the device we designed is considerable.
